# Metabolite and transcript markers for the prediction of potato drought tolerance

**DOI:** 10.1111/pbi.12840

**Published:** 2017-10-17

**Authors:** Heike Sprenger, Alexander Erban, Sylvia Seddig, Katharina Rudack, Anja Thalhammer, Mai Q. Le, Dirk Walther, Ellen Zuther, Karin I. Köhl, Joachim Kopka, Dirk K. Hincha

**Affiliations:** ^1^ Max‐Planck‐Institute of Molecular Plant Physiology Potsdam Germany; ^2^ Federal Research Centre for Cultivated Plants Julius‐Kühn Institut Institute for Resistance Research and Stress Tolerance Sanitz Germany; ^3^ VNU‐University of Sciences Thanh Xuan Hanoi Vietnam; ^4^ Present address: VIB‐UGent Center for Plant Systems Biology Technologiepark 927 9052 Ghent Belgium; ^5^ Present address: University of Potsdam Karl‐Liebknecht‐Straße 24‐25 14476 Potsdam Germany

**Keywords:** drought tolerance, machine learning, metabolite markers, potato (*Solanum tuberosum*), prediction models, transcript markers

## Abstract

Potato (*Solanum tuberosum* L.) is one of the most important food crops worldwide. Current potato varieties are highly susceptible to drought stress. In view of global climate change, selection of cultivars with improved drought tolerance and high yield potential is of paramount importance. Drought tolerance breeding of potato is currently based on direct selection according to yield and phenotypic traits and requires multiple trials under drought conditions. Marker‐assisted selection (MAS) is cheaper, faster and reduces classification errors caused by noncontrolled environmental effects. We analysed 31 potato cultivars grown under optimal and reduced water supply in six independent field trials. Drought tolerance was determined as tuber starch yield. Leaf samples from young plants were screened for preselected transcript and nontargeted metabolite abundance using qRT‐PCR and GC‐MS profiling, respectively. Transcript marker candidates were selected from a published RNA‐Seq data set. A Random Forest machine learning approach extracted metabolite and transcript markers for drought tolerance prediction with low error rates of 6% and 9%, respectively. Moreover, by combining transcript and metabolite markers, the prediction error was reduced to 4.3%. Feature selection from Random Forest models allowed model minimization, yielding a minimal combination of only 20 metabolite and transcript markers that were successfully tested for their reproducibility in 16 independent agronomic field trials. We demonstrate that a minimum combination of transcript and metabolite markers sampled at early cultivation stages predicts potato yield stability under drought largely independent of seasonal and regional agronomic conditions.

## Introduction

Potato (*Solanum tuberosum* L.) is an important food crop that is mainly grown in Europe and Asia (Haverkort and Struik, 2015). In addition, potato tubers are important as animal feed and industrial raw material (Kirkman, 2007; McGregor, 2007). Climate change scenarios predict more frequent and intense periods of drought in Europe (Jones *et al*., 2003) and many other regions of the world (IPCC, 2013). Current potato varieties are highly susceptible to drought stress, which could lead to significant tuber yield losses (Hijmans, 2003). Potato yield under drought stress is influenced by a combination of morphophysiological processes, such as photosynthesis, leaf area expansion, leaf senescence, partitioning of assimilates, tuber initiation, bulking and tuber growth (van Loon, 1981).

Thus, approaches to select genotypes with improved drought tolerance while retaining the present yield potential are of great interest. In the past, most of the breeding for drought tolerance in potato was based on selection for high yield under stress and other phenotypic traits. However, this is time‐consuming, laborious and requires field trials under drought conditions, which suffer from high weather variability (Monneveux *et al*., 2013). By contrast, marker‐assisted selection (MAS) is cheaper, faster and may be less prone to errors caused by environmental variability (Slater *et al*., 2013). The number of genotypes that have to be tested in field trials can be strongly reduced by screening breeding material for markers early during the selection cycle (Gebhardt, 2013). Molecular markers, such as transcripts or metabolites, provide an advantage because they integrate over many genes and environmental effects. This has clear advantages for complex phenotypes such as drought tolerance (Schudoma *et al*., 2012). In addition, DNA polymorphism markers may be used for the same purpose. However, while this is routinely performed in diploid crops, the use of such markers in polyploid plants such as tetraploid potato remains problematic. Nevertheless, the large‐scale application of metabolite and transcript markers in breeding will still be challenging due to the necessity of highly reproducible sampling in the field directly into liquid nitrogen to rapidly arrest all metabolic activity. Also, access to the specialized analysis platforms for qRT‐PCR and GC‐MS may be limiting.

Recent advances in ‘omics’ technologies have made the discovery of new candidate genes or metabolites for MAS possible in crops with limited or even unavailable genomic information (Gebhardt, 2013; Zabotina, 2013). However, marker candidates and the respective prediction models need to be tested with an independent set of samples to ensure robustness and generalizability (Schudoma *et al*., 2012; Zabotina, 2013). The first implementation and validation of MAS for polygenic tuber quality traits in potato was conducted by Li *et al*. (2013).

Here, we used 31 potato cultivars to identify a set of highly predictive markers for superior drought tolerance. These European potato cultivars were chosen based on their significant variation in drought tolerance (Sprenger *et al*., 2015), a prerequisite for the discovery and identification of predictive markers. Metabolite and transcript profiling was applied to leaf samples from all cultivars grown under drought stress and control conditions in field experiments. A Random Forest machine learning approach was applied to predict drought tolerance that was experimentally determined as tuber starch yield and to select optimal sets of predictive markers. These markers were tested for reproducibility on samples from independent agronomic field trials. Importantly, these markers could be used to predict drought tolerance in unstressed plants, thus making time‐consuming and expensive drought stress trials unnecessary for the breeding process.

## Results

### Characterization of drought tolerance

**Figure 1:**
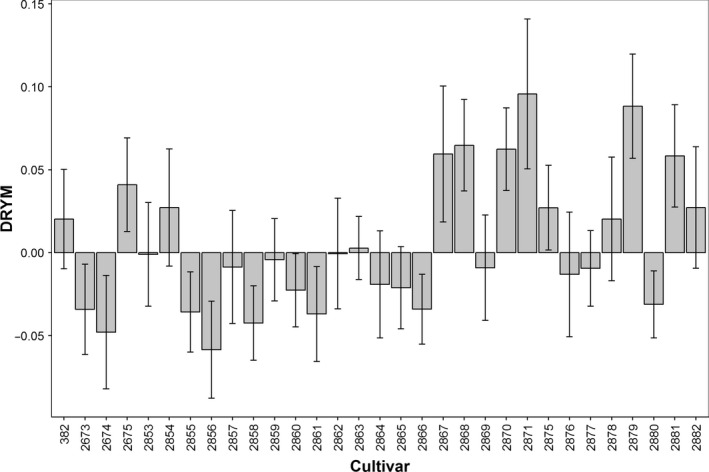
Drought tolerance of 31 potato cultivars (Table S1) based on six field experiments (F1–F5 and F7; Table S2). Drought tolerance was calculated as deviation of relative starch yield from the experimental median (DRYM). DRYM values represent mean values across experiments, and error bars represent the SE of the means. Zero indicates average tolerance, negative values indicate sensitivity, and positive values indicate tolerance.

For the quantification of drought tolerance, six independent experimental field trials on 31 potato cultivars (Table S1) were performed. They were conducted in the years 2011–2013 at three locations in Germany (Table S2). Plants were grown under optimal and reduced water supply to determine tuber starch yield and estimate drought tolerance based on the previously validated ‘deviation of relative starch yield from the experimental median’ (DRYM) index (Sprenger *et al*., 2015). The most tolerant cultivar showed a DRYM of +10% compared to the most sensitive with −6% (Figure 1). ANOVA identified cultivar as a main factor significantly influencing drought tolerance (Table 1, *P* < 0.0001).

**Table 1:** Results of ANOVA on drought tolerance in 31 potato cultivars

Source	DF	*F*	*Pr* > *F*
Cultivar	33	3.52	<0.0001
SI	1	0.02	0.8876
NSY	1	354.79	<0.0001

Degrees of freedom (DF), *F*‐statistics and error probability (*Pr* > *F*) for the effect of cultivar, stress index (SI) and starch yield under drought conditions normalized to the median starch yield under control conditions over all cultivars (NSY) on DRYM in six field experiments (DF (error) = 532).

### Selection of reference genes for qRT‐PCR

**Figure 2:**
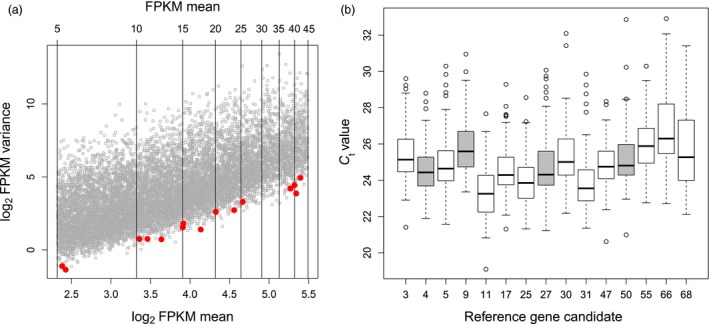
Expression plots for the selection of reference genes. (a) Relation between log_2_ FPKM mean and log_2_ FPKM variance measured by RNA‐Seq. Vertical lines indicate the expression range from 5 to 50 FPKM by an interval of 5. Selected candidates as reference genes are highlighted in red. (b) Expression of 15 candidate genes measured as *C*_t_ value by qRT‐PCR. The final selection of four reference genes is indicated in grey.

Reference genes are crucial for the accurate analysis of gene expression data by qRT‐PCR. To identify suitable reference genes that show stable expression across all cultivars, growth conditions and treatments, genes with a minimal variation in expression were selected from our previously published RNA‐Seq data (Sprenger *et al*., 2016). These data were obtained from 48 leaf samples of drought‐stressed and well‐watered plants from three glasshouse and three field trials covering four selected cultivars. Another criterion for selection as a reference gene was an expression level in an easily measurable range for qRT‐PCR. Based on these criteria, 15 candidate genes were chosen from a FPKM (fragments per kilobase of transcript per million mapped reads) interval ranging from 5 to 45 and displaying minimal variance across all samples (Figure 2a). These genes were tested by qRT‐PCR using 124 samples from glasshouse‐grown and field‐grown, well‐watered and drought‐stressed plants of all 31 cultivars (Figure 2b). Finally, four reference genes (paramyosin, ATP binding protein, β‐adaptin B and zinc finger CCCH domain‐containing protein 17) were chosen based on a minimal coefficient of variation (CV) across all tested samples (Table 2) and a nonsignificant effect of the factors cultivation type, cultivar and treatment in an ANOVA test (Table S3). To normalize the expression values for the genes of interest, the mean expression of the four reference genes was calculated.

**Table 2:** Selected reference genes for qRT‐PCR with their annotated function and coefficient of variation (CV) across 124 tested samples

Number	PGSC Gene Identifier	Functional annotation	CV
4	PGSC0003DMG400011723	Paramyosin	0.050
9	PGSC0003DMG400026492	ATP binding protein	0.056
27	PGSC0003DMG400014497	AP‐2 complex subunit β1 (β‐adaptin B)	0.064
50	PGSC0003DMG400031374	Zinc finger CCCH domain‐containing protein 17	0.064

### Selection of marker candidate genes for drought tolerance

To select suitable transcript marker candidates, we used the previously published RNA‐Seq data set (Sprenger *et al*., 2016). A subset of 298 genes exhibited higher expression in tolerant than in sensitive cultivars under control conditions in both field and glasshouse experiments. We focused on genes with higher expression in tolerant than in sensitive cultivars, because practically, the breeding process requires the identification of tolerant cultivars from a population and high transcript levels can be measured more accurately than low levels. Among those marker candidates, we identified 169 genes with a median expression level above five counts per million (CPM) across all samples from tolerant cultivars (Table S4). Finally, primer pairs targeted at 88 candidates (Table S5) were selected and transcript abundance was assessed in 202 samples from well‐watered and drought‐stressed plants of all cultivars from three independent experimental field trials (F1, F3 and F4).

**Figure 3:**
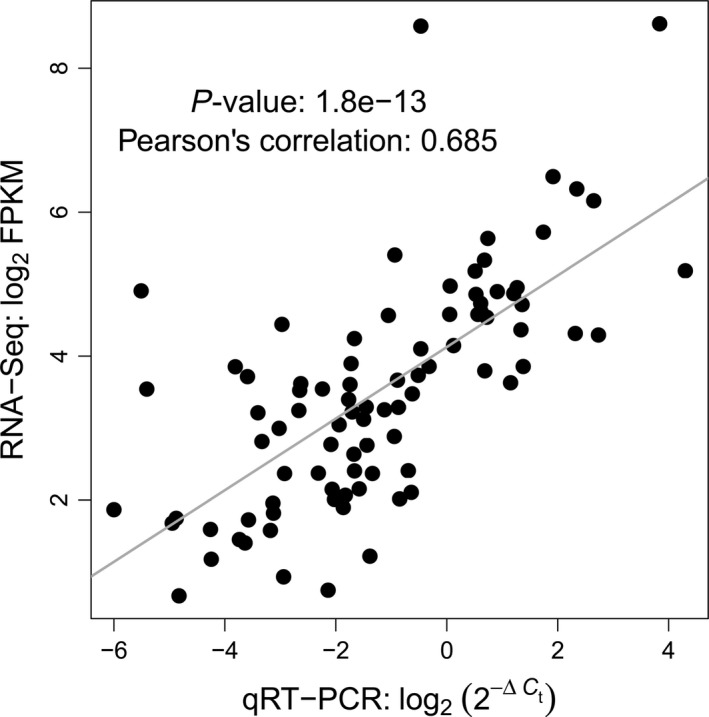
Results of qRT‐PCR using 88 selected marker candidates for drought tolerance. Correlation between gene expression measured by qRT‐PCR (log_2_($2^{-\Delta C_{t}}$)) and RNA‐Seq (log_2_ FPKM) from Sprenger *et al*. (2016).

To assess the concordance between the qRT‐PCR and RNA‐Seq data, we compared results for the 88 marker candidates from the four selected cultivars that were previously analysed by RNA sequencing (Sprenger *et al*., 2016). Gene expression measured as log_2_($2^{-\Delta C_{t}}$) by qRT‐PCR was highly significantly (*P* = 1.8E‐13, *r* = 0.685) correlated with log_2_ FPKM values gained by RNA‐Seq (Figure 3), indicating a high concordance between both methods.

### Characterization of metabolite and transcript profiles

**Figure 4:**
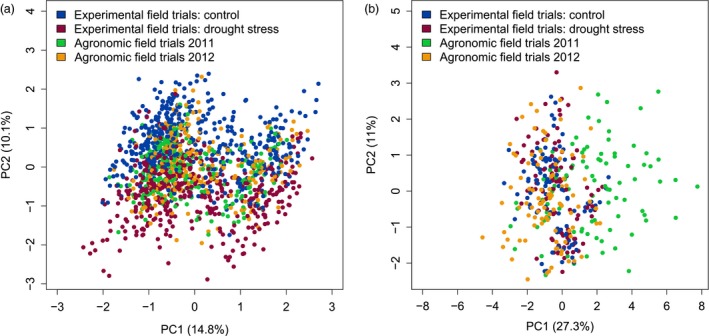
PCA scores plots of metabolite (a) and transcript (b) data of samples from field experiments and agronomic trials. PCA results indicating the difference between well‐watered control (blue) and drought‐stressed plants (red) as well as 2 years of agronomic trials (2011: green, 2012: orange) are shown for PC1 and PC2.

The full metabolome data set in the present study comprised 913 samples from five independent experimental field trials (F1–F4, F7; F5 was not used for metabolomic analysis) and 490 samples from all 16 agronomic field trials covering all 31 cultivars. Experimental field samples equally represented drought‐stressed and well‐watered conditions, while agronomic field trials reflected the variation in cultivation at eight different field sites in Germany (Table S2). In total, 115 metabolites were detected by GC‐MS across all field trials. To allow the joint analysis of data that were collected throughout 3 years, we removed systematic differences by an ANOVA‐based procedure (Lisec *et al*., 2011). The technical variance and the trial‐specific variance were reduced after applying this correction procedure (Figure S1). Subsequent principal components analysis (PCA) of the metabolite data showed a separation of samples from control and drought‐stressed plants from experimental field trials by PC2 explaining approximately 10% of the variance (Figure 4a). Samples from agronomic field trials clustered together with control samples from experimental field trials. However, the main variance of the metabolite profiles was due to the genetic differences among the cultivars, as they were separated by PC1 (14.8%) and PC3 (9.6%; Figure S2a).

The complete transcript data set consisted of 202 samples from three experimental field trials (F1, F3 and F4) and 185 samples from six agronomic field trials covering all 31 cultivars. These trials were selected from three locations, one with low yield and rainfall, second with high yield and rainfall in both years and third location with different yield and rainfall in the 2 years. Gene expression was measured for a common overlap of 43 transcript marker candidates. Part of the variance in the transcript data was due to a trial effect as PC1 (27.3%) separated the agronomic trials A6 and A8 from all other experiments (Figure S3a). In contrast to the metabolite data, the PCA scores plot of transcript data showed no separation of drought stress and control conditions (Figure 4b), in agreement with the selection criterion for these genes. Also, the genotype effect was less pronounced for the transcript compared to the metabolite data (Figure S2b).

### Metabolite markers for drought tolerance

We applied Random Forest analysis (Breiman, 2001), a machine learning method, to obtain predictive models for the drought tolerance of potato cultivars and to select metabolite markers for this trait. Training of the models was performed with metabolite data from the experimental field trials, while data from the agronomic field trials were used to test the reproducibility of the predictions. The Random Forest approach was implemented for the classification of drought tolerance into three groups: low, intermediate and high. Using the full training set including 115 metabolites as predictors, the out‐of‐bag (OOB) estimate of error rate was only 6%, corresponding to an accuracy of 94%.

**Figure 5:**
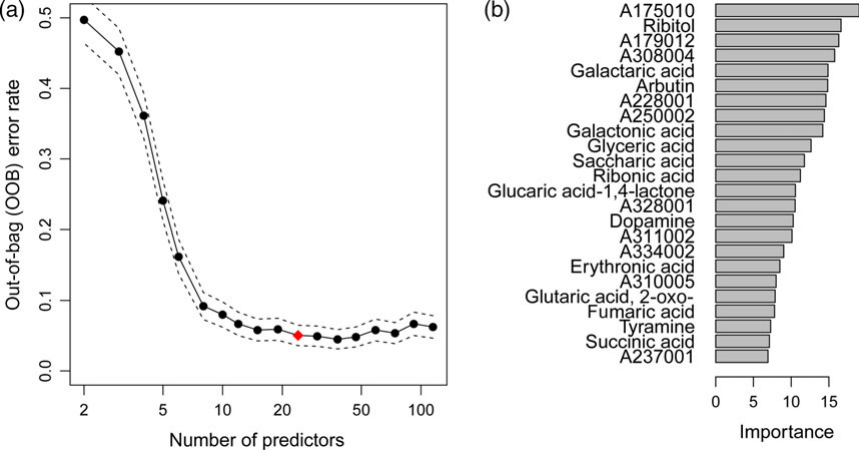
Plots illustrating the metabolite marker selection. (a) Plot of out‐of‐bag (OOB) error rate and its standard deviation (dashed lines) of the Random Forest model in relation to number of metabolite markers (predictors). The model was based on field training data. The least important predictors were eliminated successively from the model resulting in a set of 24 predictors (red diamond) according to the ‘1 SE rule’. (b) Importance of the selected 24 metabolite markers measured as mean decrease in Gini index for Random Forest models of field trial data.

In a next step, the minimal set of informative predictors in the Random Forest model was determined. Iteratively, the least important predictors were removed from the full model, and finally, the solution with the smallest number of metabolites, whose OOB error rate was within one standard error of the minimum OOB error rate of all Random Forests (‘1 SE rule’), was chosen. Variable selection resulted in a subset of 24 metabolites from a Random Forest model of the experimental field training data (Figure 5). Interestingly, the predictive accuracy was not changed compared to the full model with 115 metabolites (Table 3).

**Table 3:** Random Forest model performance for training and validation estimated by out‐of‐bag (OOB) error rate and overall accuracy. Full models based on metabolite, transcript and combined data are compared to reduced models with selected predictors

	Training (OOB error rate)		Validation (overall accuracy)	
	Full model	Reduced model	Full model	Reduced model
Metabolite data	6.02%	5.81%	91.6%	90.0%
Transcript data	8.91%	10.89%	69.7%	66.5%
Combined data	4.3%	4.3%	82.6%	77.7%

The Random Forest model estimates the importance of each predictor by the mean decrease in Gini index, where high values indicate high importance (Figure 5b). Of the 24 predictive metabolites, 10 were unidentified mass spectral tags (indicated by MST identifiers accessible at the Golm Metabolome Database, http://gmd.mpimp-golm.mpg.de/) and 10 were organic acids, such as galactaric, galactonic, glyceric and saccharic acid. Furthermore, ribitol, arbutin (4‐hydroxyphenyl‐β‐D‐glucopyranoside), dopamine and tyramine were selected as highly predictive metabolite markers.

### Transcript markers for drought tolerance

Analogous to the prediction of drought tolerance by metabolite data, gene expression data of 43 transcript marker candidates were used to build Random Forest models and to select highly informative predictors. The training of the models was performed with transcript data of three experimental field trials (F1, F3 and F4), while data of six agronomic field trials from three locations (Table S2) were used for testing the reproducibility of the predicted markers.

**Figure 6:**
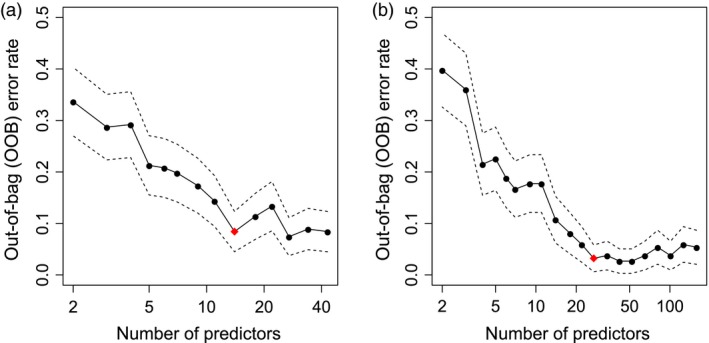
Plot of out‐of‐bag (OOB) error rate and its standard deviation (dashed lines) of the Random Forest model in relation to number of transcript markers (a). Equivalent plot of OOB error rate of the Random Forest model for combination of metabolite and transcript data (b). The models were based on field training data. The least important predictors were eliminated successively from the model resulting in a set of 14 transcripts (a) and 27 transcripts/metabolites (b), respectively (indicated by red diamond).

Using the full training set of 43 transcripts as predictors, the OOB error rate was 8.9%, slightly higher than for the model based on metabolite data. The number of informative transcript markers for prediction of drought tolerance was determined by successively eliminating the least important predictors as described for the metabolite model. This resulted in a set of 14 transcript markers as indicated by the OOB error rate in Figure 6a. As already observed for the metabolite marker model, the OOB error rate of the reduced model (10.9%) was nearly the same as for the full model including all transcripts (Table 3).

Importance of each transcript in the Random Forest model was evaluated by the mean decrease in Gini index. Table 4 lists the top 20 transcripts sorted by importance with a transcript annotated as glucosyltransferase exhibiting the highest importance. Furthermore, there are six genes in the top 20 list, which belong to the MapMan annotation bin ‘signalling receptor kinases’ (highlighted in red in Table 4). Three other genes (highlighted in blue) are classified as biotic stress‐related (BED finger‐NBS‐LRR resistance protein, bacterial spot disease resistance protein 4, TMV resistance protein N).

**Table 4:** Importance of the top 20 transcript marker candidates in Random Forest models for drought tolerance prediction based on field training data

Identifier	Functional annotation	MapMan BIN	Importance
400021019	Glucosyltransferase	26.2‐misc.UDP glucosyl and glucuronyl transferases	7.966
400031370	O‐Methyltransferase	16.2‐secondary metabolism.phenylpropanoids	5.263
400028434	Serine/threonine protein kinase, plant‐type	35.2‐not assigned.unknown	5.235
400082012	Extensin	35.2‐not assigned.unknown	5.234
400008092	Glutamyl‐tRNA (Gln) amidotransferase subunit A	26.8‐misc.nitrilases	5.219
400035714	BED finger‐NBS‐LRR resistance protein	20.1‐stress.biotic	4.988
400083025	Betaine aldehyde dehydrogenase	5.10‐fermentation.aldehyde dehydrogenase	4.551
400082023	Lipoxygenase	17.7.1.2‐hormone metabolism.jasmonate.synthesis‐degradation.lipoxygenase	4.216
400068787	Serine/threonine protein kinase, plant‐type	30.2.11‐signalling.receptor kinases.leucine rich repeat XI	4.202
400075512	Poly(ADP‐ribose) glycohydrolase	29.5‐protein.degradation	4.154
400068776	Flagellin‐sensing 2	30.2.11‐signalling.receptor kinases.leucine rich repeat XI	3.690
400071885	LRR receptor‐like serine/threonine protein kinase	30.2.11‐signalling.receptor kinases.leucine rich repeat XI	3.451
400045689	Receptor protein kinase	30.2.11‐signalling.receptor kinases.leucine rich repeat XI	3.387
400062379	Gene of unknown function	35.2‐not assigned.unknown	3.227
400004539	Glutathione S‐transferase	26.9‐misc.glutathione S transferases	3.190
400020366	Ethylene‐inducing xylanase	30.2.11‐signalling.receptor kinases.leucine rich repeat XI	3.092
400046899	TMV resistance protein N	20.1.7‐stress.biotic.PR‐proteins	3.089
400046308	Reticuline oxidase	26.8‐misc.nitrilases	3.072
400046445	Serine/threonine protein kinase, plant‐type	30.2.11‐signalling.receptor kinases.leucine rich repeat XI	3.033
400006231	Bacterial spot disease resistance protein 4	20.1.7‐stress.biotic.PR‐proteins	3.011

Variable importance was estimated by the varImp function based on the Gini index. Transcripts highlighted in grey resulted from the variable selection using the varSelRF function. Genes in the biotic stress bin are highlighted in blue, and signalling receptor kinases in red.

### Combined markers for drought tolerance

Finally, metabolite and transcript data from the three experimental field trials (F1, F3 and F4) were merged to build a combined model. The PCA scores plot of the combined data shows two main effects (Figure S3b). As already observed for metabolite data, PC1 separates samples from control and drought‐stressed plants. Additionally, samples from agronomic field trials in 2011 (A5, A6, A8) cluster together, while samples from 2012 (A9, A14, A15) are more similar to the experimental field trial samples, as already seen for the transcript data.

The combined Random Forest model including all metabolites (115) and transcripts (43) resulted in a low OOB error rate of 4.3% within the experimental field training set (Table 3). This performance was slightly improved compared to the models using either metabolite or transcript data alone for the training set, pointing to a complementing effect in the combined model. As described for the models of single data sets, the number of informative predictors was determined by successively eliminating the least important ones. This approach resulted in a set of 27 predictors (Table 5), indicated by the OOB error rate shown in Figure 6b. The error rates slightly increased with a higher number of predictors in the model, most probably due to higher noise of less informative markers. Similar to the separate transcript and metabolite models, the reduced combined model including 19 transcripts and eight metabolites showed similar performance as the full model with 158 predictors (Table 3).

**Table 5:** Importance of metabolite and transcript marker candidates in the combined Random Forest model for drought tolerance prediction based on field training data

Predictor identifier	Name	Importance
400021019	Glucosyltransferase	3.697
400031370	O‐Methyltransferase	2.908
400035714	BED finger‐NBS‐LRR resistance protein	2.704
400008092	Glutamyl‐tRNA (Gln) amidotransferase subunit A	2.610
A175010‐101	A175010‐101	2.585
400082012	Extensin	2.467
400075512	Poly(ADP‐ribose) glycohydrolase	2.383
400028434	Serine/threonine protein kinase, plant‐type	2.281
400082023	Lipoxygenase	1.933
400083025	Betaine aldehyde dehydrogenase	1.857
400068787	Serine/threonine protein kinase, plant‐type	1.750
400052517	70‐kDa subunit of replication protein A	1.742
A158004‐101	Glutaric acid, 2‐oxo‐	1.674
400068776	Flagellin‐sensing 2	1.653
400045689	Receptor protein kinase	1.590
A177001‐101	Ribonic acid	1.532
400046308	Reticuline oxidase	1.524
A179012‐101	A179012‐101	1.521
A228001‐101	A228001‐101	1.518
400062379	Gene of unknown function	1.511
400030682	Gamma aminobutyrate transaminase isoform1	1.387
400004539	Glutathione S‐transferase	1.346
A308004‐101	A308004‐101	1.278
A250002‐101	A250002‐101	1.239
A199002‐101	Galactonic acid	1.186
400027201	Acidic class II 1 3‐beta‐glucanase	1.152
400071885	LRR receptor‐like serine/threonine protein kinase	1.140

Variable importance was estimated by the varImp function based on the Gini index. Metabolites are highlighted in grey.

### Testing model reproducibility

To test the reproducibility of the prediction models trained on data from field experiments, an independent data set comprising samples from agronomic field trials was chosen. These trials were conducted at eight locations across Germany in the years 2011 and 2012 and were managed by breeding companies under realistic commercial cultivation conditions without a specific stress treatment. Metabolite profile data were obtained from all 16 trials, while transcripts were measured from six selected trials, including three from each year (A5, A6, A8, A9, A14 and A15).

As a measure of the predictive power of the Random Forest models, we used the overall accuracy (ratio of true‐positive and true‐negative cases to all). These values are summarized in Table 3 for the metabolite, transcript and combined models, using either full or reduced models as described in detail above. In general, both the full and the reduced models resulted in a similar accuracy, indicating that a set of approximately 20 predictors was sufficient for robust models in all cases. The prediction for full model validation was more accurate with metabolite data (91.6%) than with transcript data (69.7%), while the combined model showed an intermediate accuracy of 82.6%.

Tables 6 and 7 give a more detailed overview of the sensitivity (true‐positive rate) and specificity (true‐negative rate) of drought tolerance prediction using the full models. The metabolite model exhibited high sensitivity and specificity values above 90% for all tolerance classes (low, intermediate, high). In contrast, the transcript model performed with a lower sensitivity of ~80% for low and high tolerance and of only 51.5% for the intermediate tolerance class. This observation indicates that half of the intermediate samples were falsely classified as samples of either low or high tolerance.

**Table 6:** Drought tolerance prediction accuracy for all cultivars from samples taken in 16 agronomic field trials using the full Random Forest model based on metabolite data

Observed	Low	Intermediate	High
Predicted			
Low	143	5	3
Intermediate	13	157	7
High	2	10	150
Total	158	172	160
Sensitivity (%)	90.5	91.3	93.1
Specificity (%)	97.6	93.4	96.4

**Table 7:** Drought tolerance prediction accuracy for all cultivars from samples taken in six agronomic trials using the full Random Forest model based on transcript data

Observed	Low	Intermediate	High
Predicted			
Low	48	18	11
Intermediate	1	34	1
High	11	14	47
Total	60	66	59
Sensitivity	80.0	51.5	79.7
Specificity	76.8	98.3	80.2

Finally, the prediction accuracy of all models was specified for the single agronomic field trials (Table 8). The overall accuracy of the metabolite models ranged from 80.6% to 100%, indicating moderate differences between the single experiments regarding the robustness of drought tolerance prediction. However, the variability of prediction accuracy of the transcript models was larger, ranging from 45.2% to 87.1%. In particular for trials A6, A8 and A15, rather low accuracies were obtained. Mostly, the results of the combined models (82.6% accuracy) were more accurate than the transcript (69.7%), but less accurate than the metabolite models (91.6%).

**Table 8:**
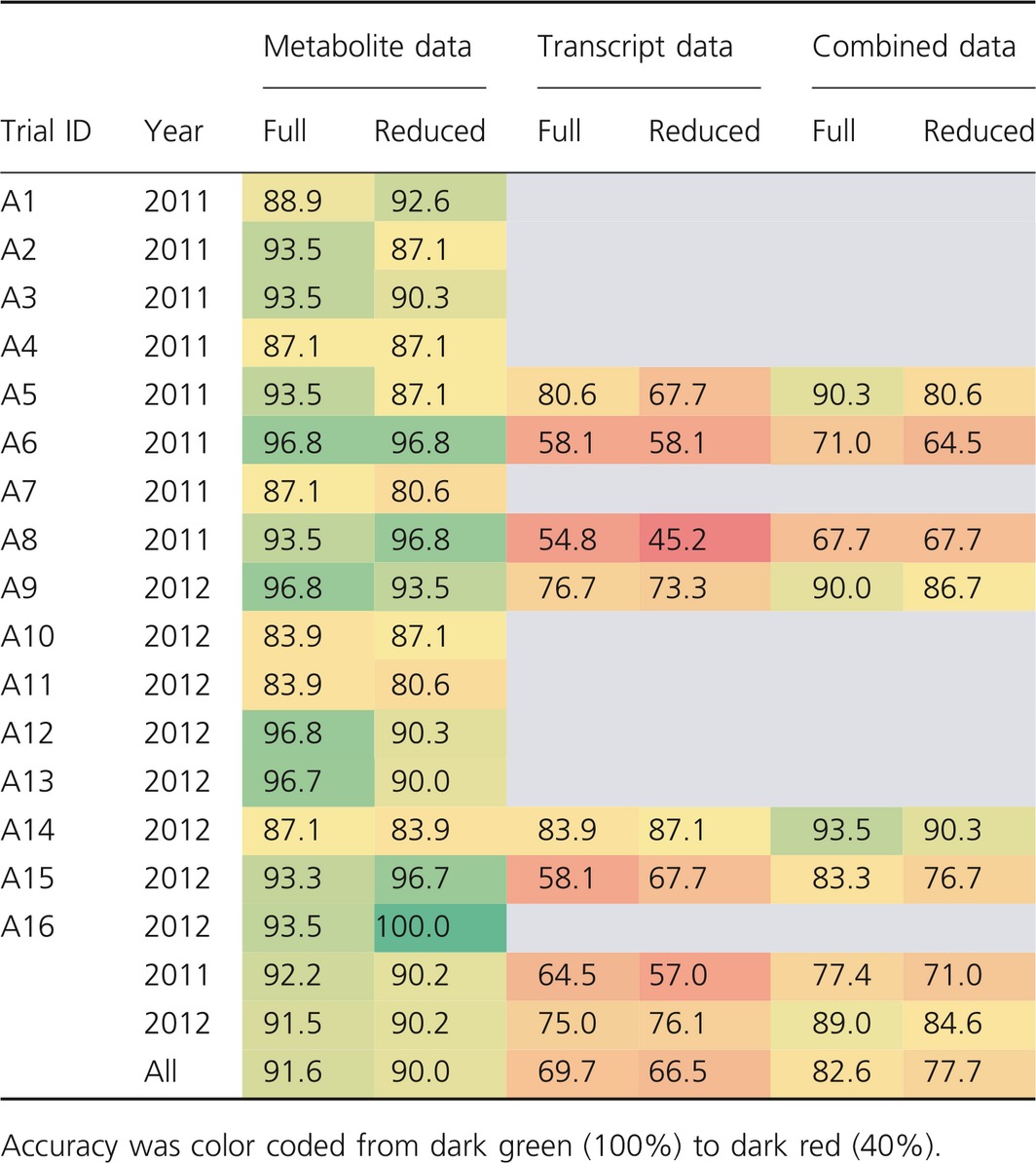
Results of model reproducibility measured as overall accuracy for the prediction of drought tolerance using agronomic field trials

## Discussion

### Characterization of drought tolerance

Drought tolerance was defined here as the deviation of relative starch yield under control and drought conditions from its experimental median (DRYM). This distinguishes drought‐tolerant from drought‐sensitive genotypes independent of their yield potential, defining drought tolerance as starch yield stability under limited water supply. DRYM was not correlated with starch yield under drought (Sprenger *et al*., 2015). It should be noted that this approach differs from the traditional breeder's approach to identify genotypes that are both high yielding and highly tolerant to drought. In the set of six experimental field trials, variation in drought tolerance was significant within the population of 31 potato cultivars and can thus be used as a basis for the discovery of drought tolerance markers.

### Selection of reference genes for qRT‐PCR

Quantification of gene expression by qRT‐PCR requires adequate reference genes, which show stable expression across the diversity of cultivars and growth conditions (Bustin *et al*., 2009; Remans *et al*., 2014). Hruz *et al*. (2011) showed that no single gene is universally stably expressed. Therefore, reference genes have to be validated for the particular biological context. Based on a previously published RNA‐Seq data set (Sprenger *et al*., 2016), we selected four genes whose expression was most stable across all 31 cultivars under control and drought stress conditions. These genes also exhibit a very low coefficient of variation (CV) in a RNA‐Seq data set of 6386 constitutively expressed genes for 32 tissues and growth conditions from the doubled monoploid *S. tuberosum* group Phureja clone DM1‐3 516R44 (Massa *et al*., 2011). This indicates that these validated reference genes will also be useful for other studies in potato.

### Selection of marker metabolites and transcripts

For the discovery and validation of meaningful and robust markers, we sampled the first fully expanded leaf from plants grown in several independent field trials at different locations and during several years. The precise definition of the sampling material and the sampling time in terms of developmental stage and time during the day minimized the confounding variation. Further confounding factors specific to GC‐MS profiling were successfully eliminated by an ANOVA‐based data preprocessing. There was only a slight separation between drought‐stressed and control samples from experimental field trials, while the latter tended to cluster with samples from agronomic field trials. However, most of the metabolic variance was due to genetic differences among the cultivars.

To select candidate genes as drought tolerance markers, we used a nontargeted approach based on RNA‐Seq transcript profiles from two tolerant and two sensitive test cultivars (Sprenger *et al*., 2016). We tested the expression of 88 of these genes in a set of 31 cultivars. The highly significant correlation between gene expression measured by qRT‐PCR and RNA‐Seq indicated a high degree of consistency between these methods that had also been reported in humans, maize and potato (Gao *et al*., 2013; Li *et al*., 2010, 2015; Roberts *et al*., 2011).

Our approach of preselecting putative markers from a genome‐wide analysis was a critical step, because only this untargeted global transcriptome analysis allows the discovery of novel marker candidates that can then be tested in a targeted qRT‐PCR approach. Similar to this strategy, other studies used preselection to enable high‐throughput screening of large sample sets. For example, 184 candidate genes for flesh colour of potato tubers were selected from microarray experiments (Kloosterman *et al*., 2010), and subsequently, candidates were successfully checked for an association with the trait of interest by qRT‐PCR.

Another strategy for the identification of marker candidates is based on a significant genotype × environment interaction. From a microarray study on four rice cultivars with contrasting drought tolerance (Degenkolbe *et al*., 2013), 46 (of 108) potential markers were selected and tested by qRT‐PCR in 21 cultivars with varying drought tolerance. Gene expression levels of 28 of these candidates correlated significantly with performance parameters under drought stress. However, this approach does not involve a prediction model and therefore does not allow the selection of optimal marker combinations. Also, such markers are identified by their differential stress responsiveness, while the marker candidates identified here were not differentially expressed in response to drought stress. Genes whose expression is significantly associated with drought tolerance already under control conditions are better suited for large‐scale breeding programmes, as they can be used without the imposition of environmental stress conditions. In the field, such conditions are difficult to control and may vary significantly from year to year due to interactions with other abiotic and biotic factors.

### Prediction models for drought tolerance

To identify the most informative markers and to generate robust prediction models from large omics data sets, machine learning methods are frequently applied (Schudoma *et al*., 2012). Here, we used Random Forest models to predict drought tolerance classes and identify molecular markers. Random Forest shows similar performance as other classification methods (SVM, LDA, PLS and KNN) or even outperforms them using metabolomics data (Chen *et al*., 2013; Nam *et al*., 2009; Wu *et al*., 2003). It is suitable for multiclass problems and allows the selection of small sets of markers while maintaining predictive accuracy (Díaz‐Uriarte and Alvarez de Andrés, 2006). Using the full training set resulted in surprisingly high prediction accuracies of 94% and 91% for metabolite‐ and transcript‐based classification models, given the approximately 2 months time lag between sampling for marker analysis and tuber harvest for the analysis of starch yield. Korn *et al*. (2010) established PLS models to predict the freezing tolerance of different Arabidopsis genotypes from metabolite composition with high accuracy (82%–87%). The performance of Random Forest models for the prediction of late blight resistance and tuber yield of potato by peptide markers was comparable with accuracy values of 78% and 76% (Chawade *et al*., 2016). Also, in rice, the prediction accuracy of PLS regression using metabolite markers ranged from 86% to 98% for the multigenic traits yield, heading date and plant height (Dan *et al*., 2016).

Even though the accuracy was already above 90% for the single‐variable models, the performance of the combined Random Forest model was still slightly higher. Similarly, the combination of metabolic and genetic markers leads only to moderate improvement of the prediction of hybrid biomass in Arabidopsis by PLS regression (Gärtner *et al*., 2009; Steinfath *et al*., 2010a).

The application of high‐throughput methods results in a large number of variables in prediction models often derived from small numbers of samples, possibly leading to a high degree of multicollinearity and bearing the risk of overfitting (Jannink *et al*., 2010). As Random Forests employ a built‐in cross‐validation, the risk of overfitting was already reduced by the choice of classification method and the reduction in variables included in the prediction models further reduced this risk. At the same time, this reduced model complexity improves applicability for breeding programmes. Remarkably, we observed only marginal differences in performance between full models and models where the number of markers was reduced by 70%–80%. As Random Forests contain a random selection of training samples and associated identification of informative variables, the perils of erroneously assigning low importance to variables that correlate with others may also be reduced as correlated variables will have similar chances of being selected in the different random trials. Comparable results were reported by Dan *et al*. (2016), Steinfath *et al*. (2010a) and Gärtner *et al*. (2009), while Korn *et al*. (2010) even observed a slight increase in the predictive power for the optimal selection of metabolites. The prediction of potato chip quality was successfully applied on a segregating breeding population confirming two sugars as metabolite markers (Steinfath *et al*., 2010b).

Most of the informative metabolite markers for drought tolerance are organic acids (e.g. galactaric, galactonic, glyceric and saccharic acid) in addition to 10 unidentified mass spectral tags. Eight of these 24 metabolites (arbutin, tyramine, fumaric acid, galactonic acid, ribitol, A179012, A228001 and A237001) were also present in significantly different amounts in tolerant and sensitive cultivars under control conditions in field trials with four cultivars in our previous study (Sprenger *et al*., 2016). Arbutin has not only been linked to drought, but also to desiccation tolerance and pathogen resistance, as discussed in detail previously (Sprenger *et al*., 2016). Mane *et al*. (2008) reported higher concentrations of 2‐oxo‐glutaric acid in a drought‐tolerant than in a sensitive Andean potato genotype. Interestingly, a study of rice cultivars discovered a positive correlation between levels of galactaric acid and drought tolerance, but negative correlations for erythronic and galactonic acid (Degenkolbe *et al*., 2013). In addition, 2‐oxo‐glutaric acid and succinic acid are positively associated with high night temperature (HNT) tolerance of rice, whereas saccharic acid shows a negative correlation (Glaubitz *et al*., 2017).

Further highly predictive markers were the catecholamines dopamine and tyramine that are involved in many aspects of plant growth and development. They may affect the regulation of plant hormones and carbohydrates, protect plants against pathogens and influence nitrogen detoxification (Kulma and Szopa, 2007). Their synthesis is up‐regulated by stress conditions, such as wounding, ABA treatment and drought in potato leaves (Świędrych *et al*., 2004; Szopa *et al*., 2001).

Among the identified most informative transcript markers, six genes are related to signalling and receptor kinases. Additionally, we identified marker transcripts annotated as pathogen defence related that have already been suggested as potential drought tolerance markers previously (Sprenger *et al*., 2016). Interestingly, most of the top 20 transcript markers also showed higher abundance under different biotic stress treatments in *S. tuberosum* group Phureja clone *DM1‐3 516R44* (Massa *et al*., 2011) and upon BABA treatment of potato cultivar Desiree (Bengtsson *et al*., 2014), which was also included in the population investigated here. This further substantiates our previous conclusion (Sprenger *et al*., 2016) that constitutive differences in metabolite and transcript levels between tolerant and sensitive potato cultivars indicate interactions of drought tolerance and pathogen resistance. Here, cross‐talk between abiotic and biotic stress signalling may be explained by expression of partially overlapping sets of genes (Fujita *et al*., 2006; Rejeb *et al*., 2014), leading, for example, to enhanced resistance to the fungus *Botrytis cinerea* in tomato under drought stress (Achuo *et al*., 2006; Mohr and Cahill, 2003). Also, cucumber mosaic virus infection improved drought and freezing tolerance of beet and tobacco plants (Xu *et al*., 2008).

### Reproducibility of model predictions

Both the transcriptome and metabolome are highly dynamic and change in response to varying environmental conditions. To be practically useful, the predictive power of molecular biomarkers should be independent of environmental factors. We tested the prediction models derived from experimental field trials using a wide range of agronomic field trial conditions. The resulting gene expression and metabolite profiling data were used to predict the drought tolerance of our study population. The metabolite model successfully predicted drought tolerance of the cultivars from samples taken in all 16 agronomic field trials. Constraining the model to the reduced set of 24 markers resulted in similar accuracy values.

The application of the transcript model trained on samples from three experimental field trials led to a high prediction accuracy for six selected agronomic field trials, although prediction accuracy was lower compared to the metabolite model. However, the selection of transcript markers was based on only four cultivars (Sprenger *et al*., 2016), while metabolite markers were measured for all 31 cultivars. In addition, the transcript model was only built on samples from three experimental field trials, compared to the five trials for the metabolite model. Thus, including more cultivars for the selection of transcript markers and more experimental data for the model training might further improve the predictive performance.

Steinfath *et al*. (2010b) confirmed the predictive power of metabolite markers for the susceptibility to black spot bruising and potato chip quality by comparison with an independent data set, while DNA‐based markers for potato tuber quality showed only limited reproducibility (Li *et al*., 2013). In general, there still is a lack of studies that incorporate the challenging but important step of testing marker reproducibility on independent data to avoid overly optimistic predictions of marker efficiency.

Overall, our study demonstrates the feasibility of predicting drought tolerance from a small number of transcript or metabolite markers. In future, validation of our prediction models with additional genotypes could be used to check the transferability to independent populations to facilitate potato breeding by MAS (Slater *et al*., 2014). Finally, the successful evaluation with independent agronomic field trials demonstrates the high robustness of the prediction models in diverse environments. Thus, the requirement for expensive controlled drought stress experiments may be reduced by early prediction of drought tolerance using transcript or metabolite markers.

## Experimental procedures

### Plant material and stress treatment

Six experimental field trials (F1–F5, F7, Table S2) were conducted using 31 potato (*Solanum tuberosum* L.) cultivars as described by Sprenger *et al*. (2015). In F1 and F3, control plants were drip‐irrigated from the top of the ridges with 10 L/m^2^ water during the night when plants showed signs of decreased turgor at noon. Drought‐stressed plants were irrigated when they showed visible wilting 2 h after sunrise. F4 and F7 were carried out under a rain‐out‐shelter where drought stress was applied by stopping watering at the beginning of emergence of tubers. In F2 and F5, two control blocks were irrigated when soil water content fell below 50% field capacity. Two blocks were irrigated at 30% field capacity and two were not irrigated.

For the agronomic field trials, all 31 cultivars were cultivated in duplicate plots on eight sites managed by breeding companies in Germany in 2011 and 2012 under their routine conditions. For details on the location, duration and water supply, see Table S2

### Sampling and phenotypic characterization

Approximately 65 days after planting, leaf samples were harvested as described in detail previously (Sprenger *et al*., 2016). Two leaflets of the first fully developed compound leaf were immediately frozen in liquid nitrogen and stored at −80 °C until use. Subsequently, all samples were homogenized using a cryogenic grinding robot (Labman Automation, http://www.labman.co.uk). Tubers were harvested after approximately 100 days of cultivation. The stress index (SI) of Experiment *i* was calculated from the average starch yield in Experiment *i* for all cultivars under drought (*T*_d_) or control treatment (*T*_c_) as follows:

$${\text{SI}}_{i}=1-\left(\frac{\text{mean}\left({\text{Starchyield}}_{E_{i},T_{d}}\right)}{\text{mean}\left({\text{Starchyield}}_{E_{i},T_{c}}\right)}\right).$$

DRYM was calculated as described recently (Sprenger *et al*., 2015).

### GC‐MS analysis of primary metabolites

Metabolite profiling was performed as in Sprenger *et al*. (2016) by gas chromatography coupled to electron impact ionization time of flight‐mass spectrometry (GC/EI‐TOF‐MS). Plant material from two to four replicate plants per cultivar and condition was pooled. The number of biological replicates per cultivar and condition is given in Table S6. Metabolite intensities were log_10_‐transformed to approximate normal distribution. ANOVA was applied within the R software using genotype, measurement batch and sample sequence as well as averaged original intensity (response) of all annotated analytes as factors. Systematic differences due to the three latter factors were removed by ANOVA to enable joint analysis of individual measurement campaigns (Lisec *et al*., 2011).

### qRT‐PCR analysis of gene expression

Homogenized leaf material of two to four replicates per cultivar and treatment was pooled to approximately 100 mg in 96‐well plates (Collection Microtubes, Qiagen, Hilden, Germany). Total RNA was isolated using a TRIzol protocol based on the ‘single step’ method (Chomczynski and Sacchi, 1987). For extraction, 500 μL TRIzol reagent (Ambion, Life Technologies, Carlsbad, CA) and 200 μL chloroform were used. RNA was precipitated by adding 125 μL isopropanol and 125 μL 2 m NaCl. After washing the pellets twice with 70% ethanol, they were resuspended in 50 μL RNase‐free water. RNA concentration and integrity were determined with the NanoDrop 1000 UV‐VIS spectrometer (Thermo Scientific, Wilmington, DE) and on a 1.7% (w/v) agarose gel. Four microgram of each RNA sample was further treated with RapidOut DNA Removal kit (Thermo Scientific). Absence of genomic DNA contamination was confirmed by quantitative PCR using an intron‐specific primer pair for Rubisco small subunit (Table S5). One microgram of total RNA was transcribed into cDNA by SuperScript III Reverse Transcriptase (Thermo Scientific). The quality and yield of cDNA were assessed by qRT‐PCR with primers for the 5′ and 3′ ends of GAPDH (Table S5). GAPDH primer pair version 1 (Degenkolbe *et al*., 2005) was used for all cultivars, except for cultivars 2868 and 2875, for which version 2 was used.

Primers to detect candidate marker transcripts were designed using the Primer3 online tool (http://primer3.wi.mit.edu/). Primer sequences were checked against the PGSC *S. tuberosum* group Phureja clone DM1‐3 516R44 transcript reference sequences (v3.4) using an online search tool (http://solanaceae.plantbiology.msu.edu/integrated_searches.shtml). All primer pairs were tested against cDNA from all 31 cultivars under both control and drought stress conditions prior to the actual experiments. Melting curves of the PCR products were inspected to ensure that only one unique product was produced.

Finally, qRT‐PCR was performed in 384‐well plates with an ABI PRISM 7900 HT Sequence Detection System (Applied Biosystems, Foster City, CA). Reactions contained 2.5 μL Power SYBR Green reagent (Applied Biosystems), 0.5 μL cDNA (diluted fivefold) and 2 μL of 0.5 μm primers in a total volume of 5 μL. A pipetting robot Evolution P3 (Perkin Elmer, Zaventem, Belgium) was used to dilute and dispense the primers and to add sample mix (cDNA and SYBR Green). Cycle threshold (*C*_t_) values for marker candidate genes were normalized by subtracting the mean *C*_t_ of four reference genes that were included on each plate. Relative gene expression was calculated as $2^{-\Delta C_{t}}$ and log_10_‐transformed to approximate normal distribution. Primer sequences are listed in Table S5, including primers for the four reference genes. Expression values for tested reference genes and normalized expression values for all candidate genes are given in Tables S3 and S7, respectively.

### Predictive model of drought tolerance

Drought tolerance of the 31 cultivars was assessed by DRYM calculated for each cultivar and trial by subtracting the median of the relative starch yield of each trial from the relative starch yield for the respective cultivar and trial (Sprenger *et al*., 2015). DRYM values of six independent field experiments (F1–F5, F7) were averaged and classified into three levels (high, medium and low) based on tertiles of the probability distribution.

Missing values in the metabolite (5.5%) and transcript (2.2%) data were estimated by PCA using the Nipals method (R‐package *pcaMethods*; Stacklies *et al*., 2007).

For drought tolerance prediction by Random Forest models, data from the experimental field trials were used as a training set, while samples of 16 independent agronomic field trials were used for metabolite model validation. Samples from six agronomic field trials were used for transcript model validation. The training set included 913 samples for metabolite data (115 predictors) and 202 samples for transcript data (43 predictors). The validation set was comprised of 490 samples for metabolite data and 185 samples for transcript data.

Random Forest is a machine learning method that uses a collection of unpruned decision trees, each of which is built on a bootstrap sample of the training data using a randomly selected subset of predictors (Breiman, 2001). In this study, the R‐package *randomForest* (Liaw and Wiener, 2002) was used to implement the prediction model with the two main parameters ntree = 1000 and mtry set to default (=$\sqrt{p}$, where p is the number of predictors). Variable importance was estimated by the varImp function and is based on the Gini index. The number of predictors was reduced by the varSelRF function of the R‐package *varSelRF* (Díaz‐Uriarte, 2007) to minimize the out‐of‐bag (OOB) error rate (a measure for the prediction error that uses bootstrap aggregating, also called bagging). Iteratively, the least important variables were removed from the full model, and finally, the solution with the smallest number of predictors, whose OOB error rate was within one standard error of the minimum OOB error rate of all Random Forests (‘1 SE rule’), was chosen. Precision measures of the models (accuracy, sensitivity and specificity) were obtained by the confusionMatrix function from the R‐package *caret* (Kuhn, 2008).

## Conflict of interest

The authors declare no conflict of interest.

## Supporting information


**Figure S1** PCA scores plot of metabolite data of samples from experimental and agronomic field trials before (a) and after (b) an ANOVA‐based correction procedure.

**Figure S2** PCA scores plot of metabolite (a) and transcript (b) data of samples from experimental and agronomic field trials.

**Figure S3** PCA scores plot of transcript (a) and combined (b) data of samples from three field (circles: control, squares: drought stress) and six agronomic trials (diamonds: 2011, triangles: 2012).

**Table S1** Cultivar identifier, cultivar name, breeding company and drought tolerance expressed as the deviation of relative starch yield from the experimental median (DRYM) for the 31 potato cultivars used in this study.

**Table S2** Description of the field trials (F1–F5, and F7) and cultivation in agricultural environments (type A) of 31 potato cultivars.

**Table S3** List of 298 transcript marker candidates including the subsets of 169, 88 and 43 transcript marker.

**Table S4** List of qRT‐PCR primer sequences that were used for transcript marker validation and quality checks.

**Table S5** List of the expression values of 15 reference candidate genes investigated in 124 samples.

**Table S6** List of the expression values of marker candidate genes investigated in the samples used in this study.

**Table S7** Corrected normalized metabolome data of experimental field and agronomic field trials, F1–F4, F7 and A1–A16.
