# Supplementary material for: Metabolite and transcript markers for the prediction of potato drought tolerance
Source: Plant Biotechnol J. 2017 Oct 17;16(4):939–50. doi: 10.1111/pbi.12840 (PMC5866952; doi:10.1111/pbi.12840)
Supplement: Supplementary file 1 — Figure S1 PCA scores plot of metabolite data of samples from experimental and agronomic field trials before (a) and after (b) an ANOVA‐based correction procedure. [file PBI-16-939-s005.pdf]

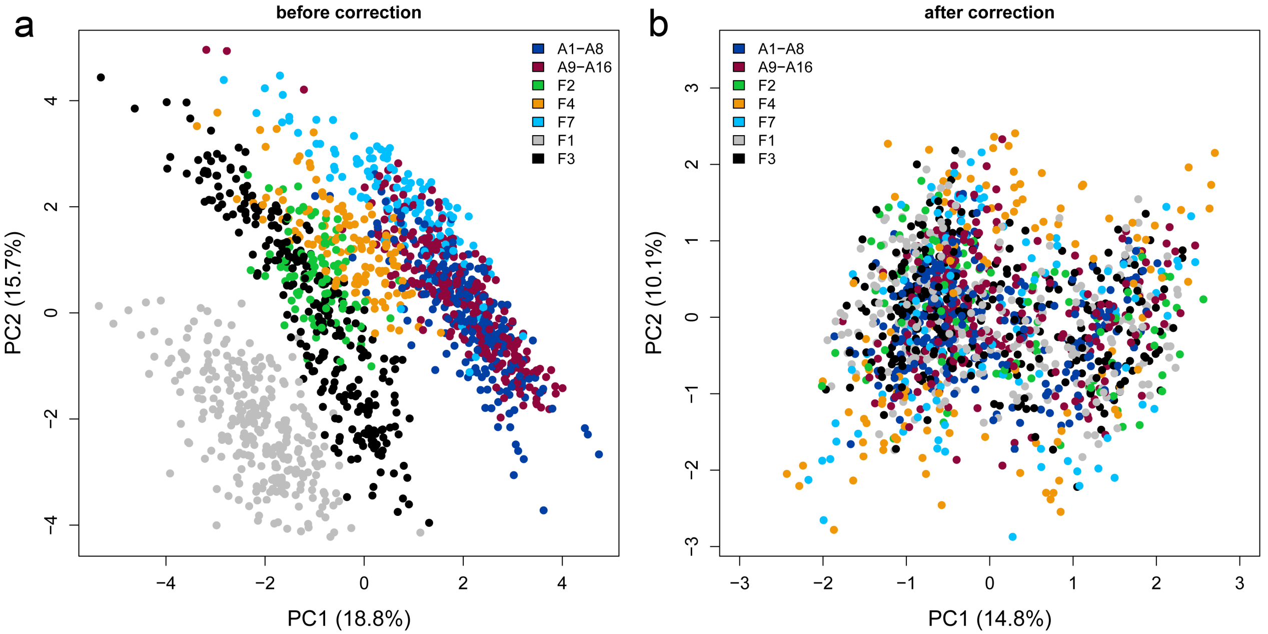

Supplemental Figure 1: PCA scores plot of metabolite data of samples from experimental and agronomic field trials before (a) and after (b) an ANOVA-based correction procedure. PCA results indicating the difference between experimental (F1-F4, F7) and agronomic field trials (A1-A16) are shown for PC1 and PC2.
