# Supplementary material for: Metabolite and transcript markers for the prediction of potato drought tolerance
Source: Plant Biotechnol J. 2017 Oct 17;16(4):939–50. doi: 10.1111/pbi.12840 (PMC5866952; doi:10.1111/pbi.12840)
Supplement: Supplementary file 2 — Figure S2 PCA scores plot of metabolite (a) and transcript (b) data of samples from experimental and agronomic field trials. [file PBI-16-939-s008.pdf]

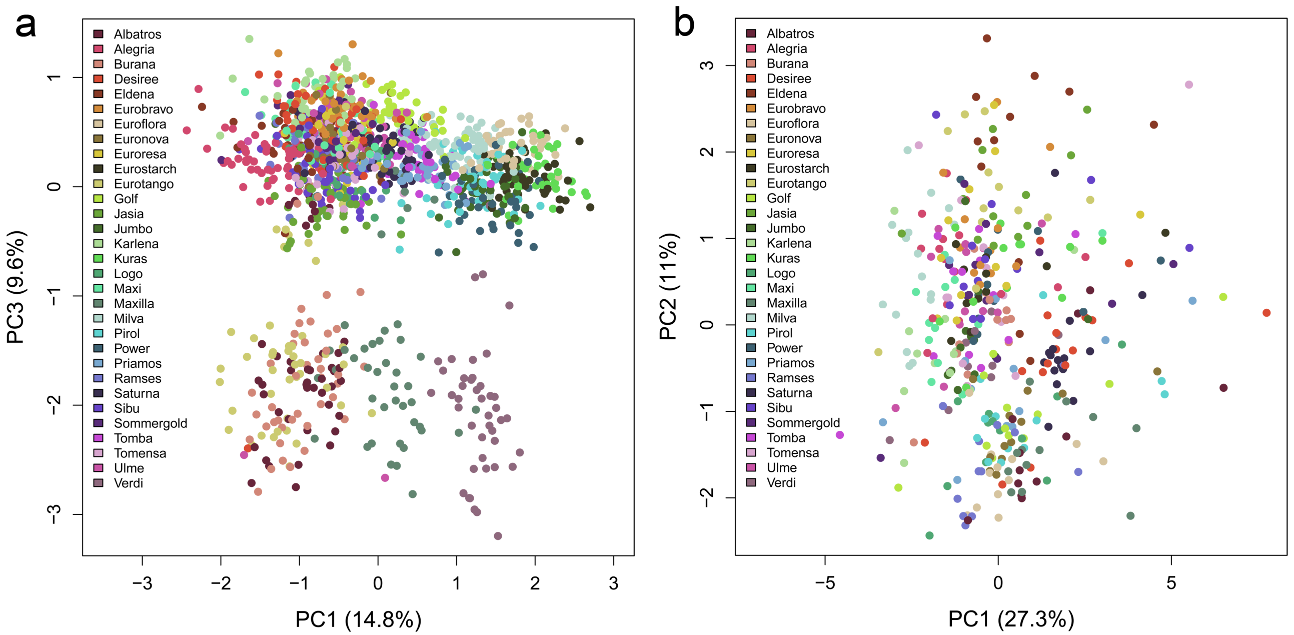

Supplemental Figure 2: PCA scores plot of metabolite (a) and transcript (b) data of samples from experimental and agronomic field trials. PCA results indicating the difference between 31 cultivars are shown for PC1 and PC3 (a) or PC1 and PC2 (b).
