# Supplementary material for: Metabolite and transcript markers for the prediction of potato drought tolerance
Source: Plant Biotechnol J. 2017 Oct 17;16(4):939–50. doi: 10.1111/pbi.12840 (PMC5866952; doi:10.1111/pbi.12840)
Supplement: Supplementary file 3 — Figure S3 PCA scores plot of transcript (a) and combined (b) data of samples from three field (circles: control, squares: drought stress) and six agronomic trials (diamonds: 2011, triangles: 2012). [file PBI-16-939-s007.pdf]

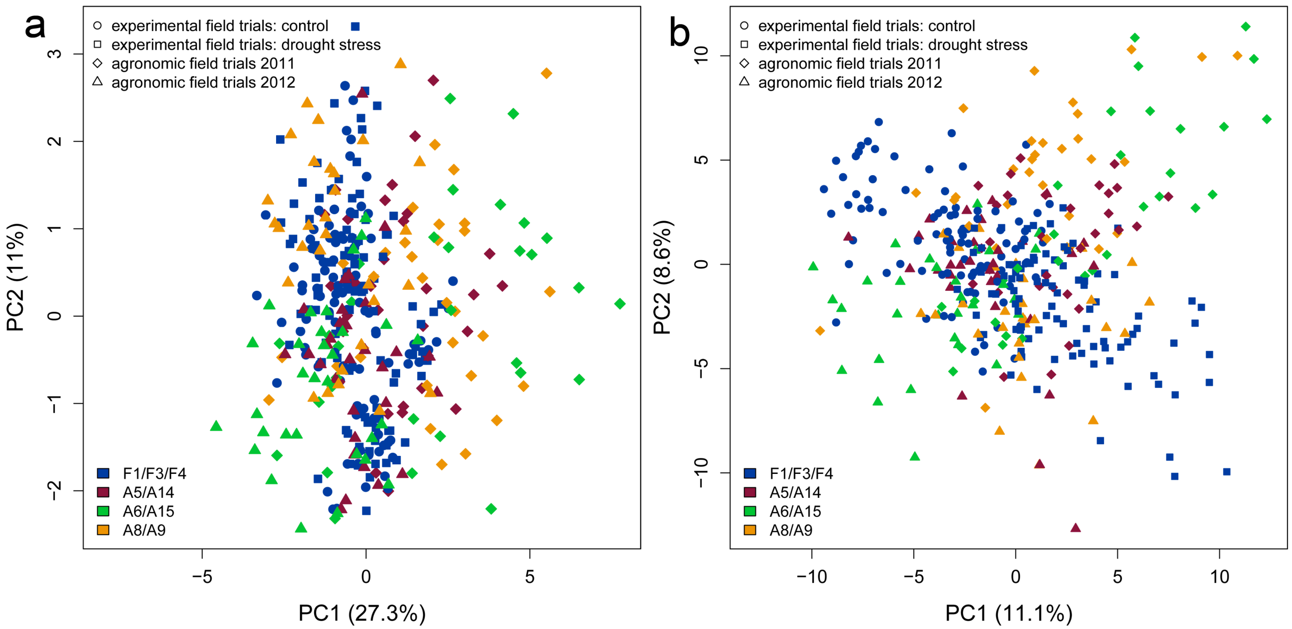

Supplemental Figure 3: PCA scores plot of transcript (a) and combined (b) data of samples from three field (squares: control, diamonds: drought stress) and six agronomic trials (circles: 2011, triangles: 2012). PCA results indicating the difference between these nine trials are shown for PC1 and PC2. See Supplemental Table S2 for additional information about experimental (F) and agronomic (A) field trials.
